# Supplementary figures and images for: Kicking sleepers out of bed: Macrophages promote reactivation of dormant Cryptococcus neoformans by extracellular vesicle release and non-lytic exocytosis
Source: PLoS Pathog. 2023 Nov 30;19(11):e1011841. doi: 10.1371/journal.ppat.1011841 (PMC10715671; doi:10.1371/journal.ppat.1011841)

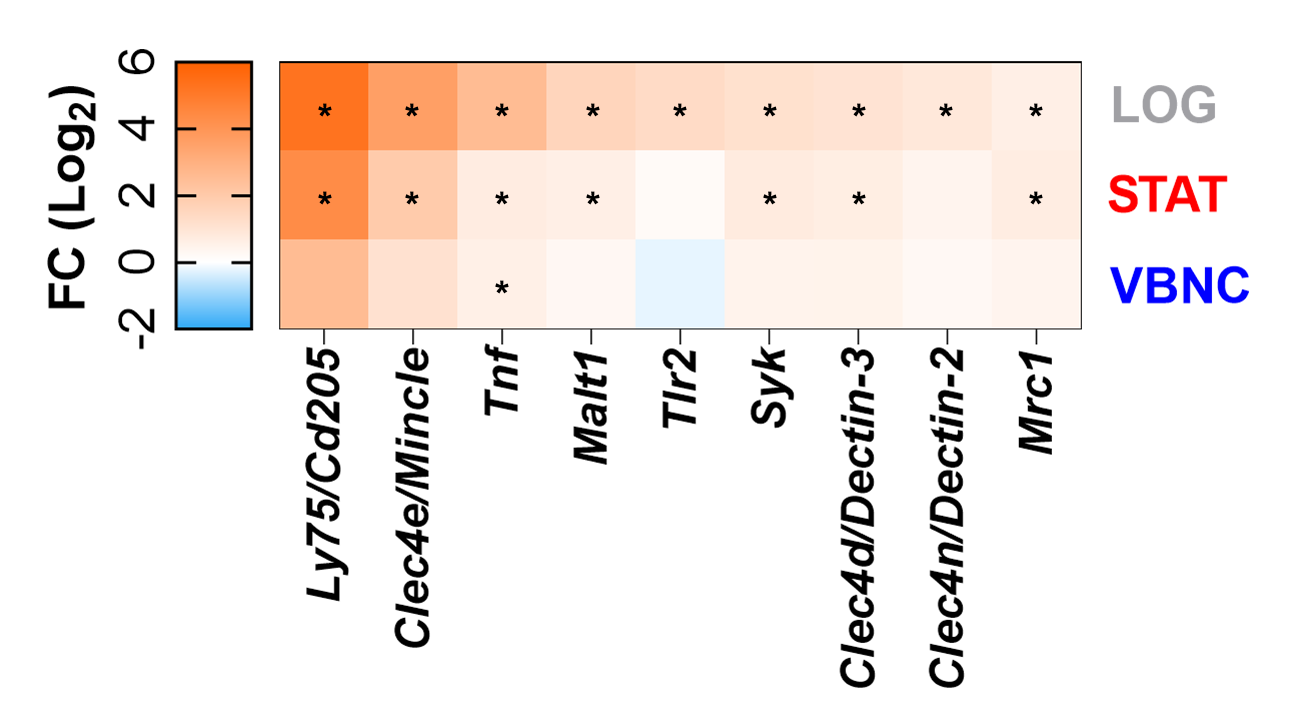

Supplement: S1 Fig — (TIF) [file ppat.1011841.s001.tif]

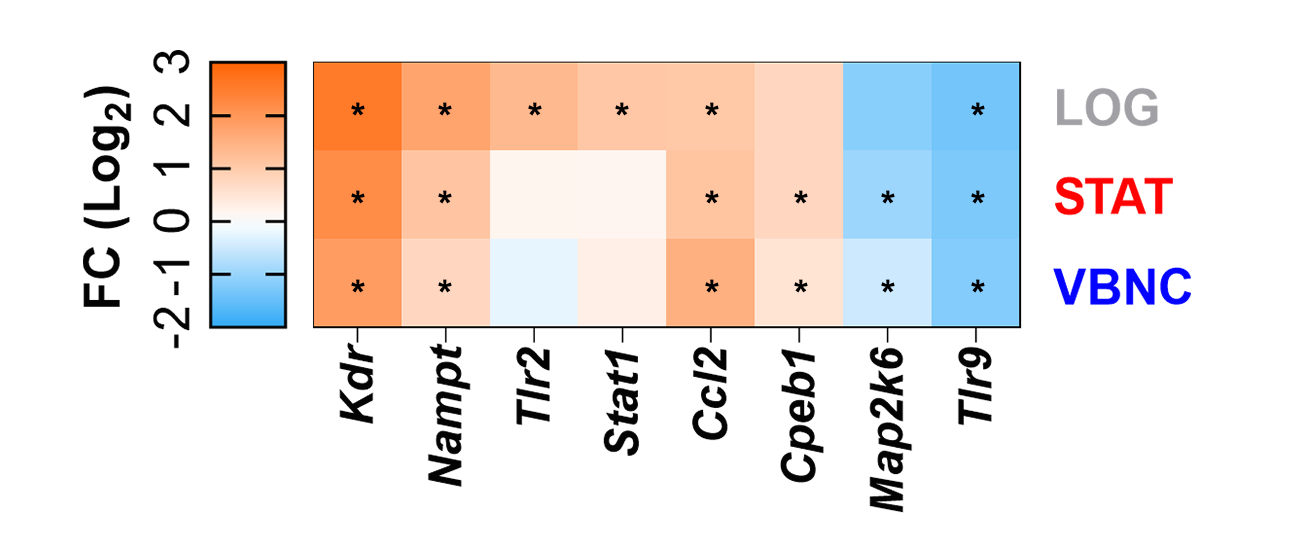

Supplement: S2 Fig — (TIF) [file ppat.1011841.s002.tif]

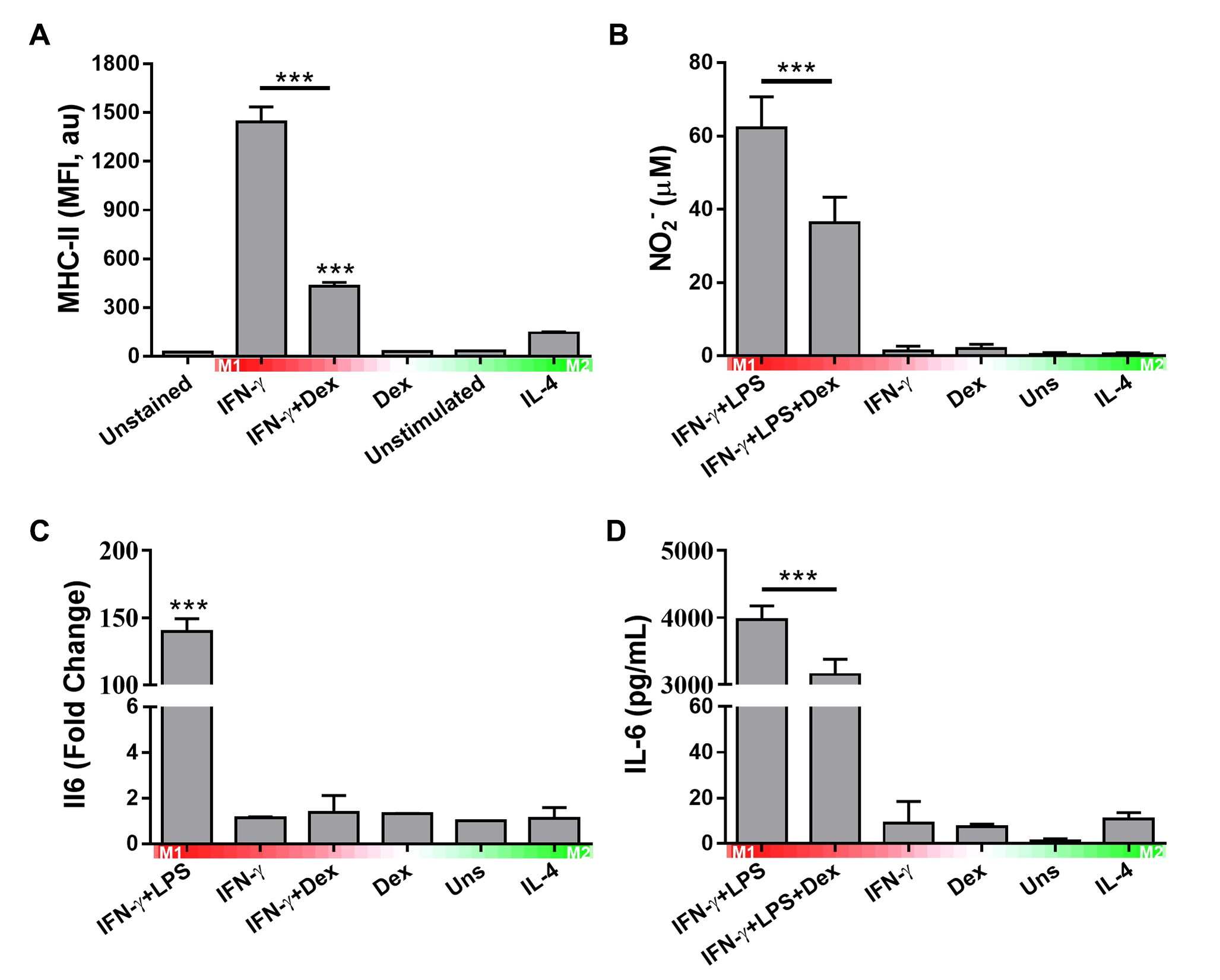

Supplement: S3 Fig — (A) Median fluorescence intensity (MFI; arbitrary units, a.u.) of BMDMs after indicated treatments showing increased MHC-II expression upon IFN-ɣ stimulation, which is attenuated by concomitant administration of dexamethasone (Dex). (B) Detection of increased levels of nitrite in the supernatant of BMDMs stimulated with IFN-ɣ+LPS, which are damped by the co-addition of dexamethasone. Co-stimulation with IFN-ɣ and LPS enhanced transcription (C) and secretion of IL-6 (D), as assessed by real-time PCR and ELISA assay, respectively. Addition of dexamethasone decreased IL-6 release. Analyzes were conducted after 24 h of treatment. Il-6 gene transcription was normalized against the constitutive gene GAPDH and expressed as fold change. Data from 1 representative experiment out of 2. ***p<0.001, between the indicated groups or compared to unstimulated control group (uns). (TIF) [file ppat.1011841.s003.tif]

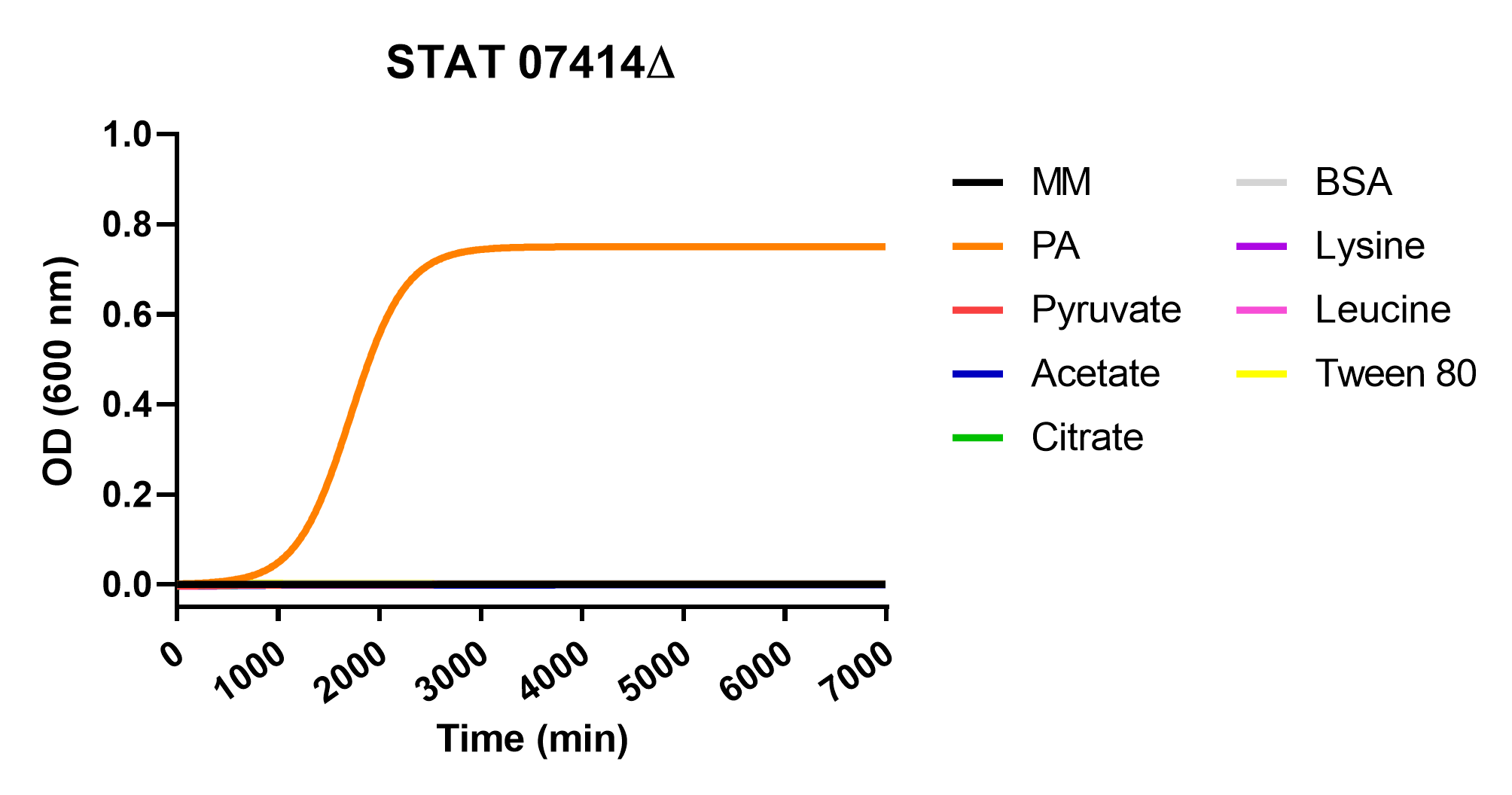

Supplement: S4 Fig — (A) C. neoformans 07414Δ mutant strain was incubated in minimal medium (MM) supplemented with the CoA precursor PA, or with the following primary/secondary precursors of acetyl-CoA: pyruvate, acetate, citrate, ketogenic amino acids (lysine and leucine), tween 80 (as a source of fatty acids), as well as bovine serum albumin (BSA). Growth was solely observed in the presence of PA. Data are means from 1 representative experiment out of 3, conducted with 3–5 technical replicates each. (TIF) [file ppat.1011841.s004.tif]

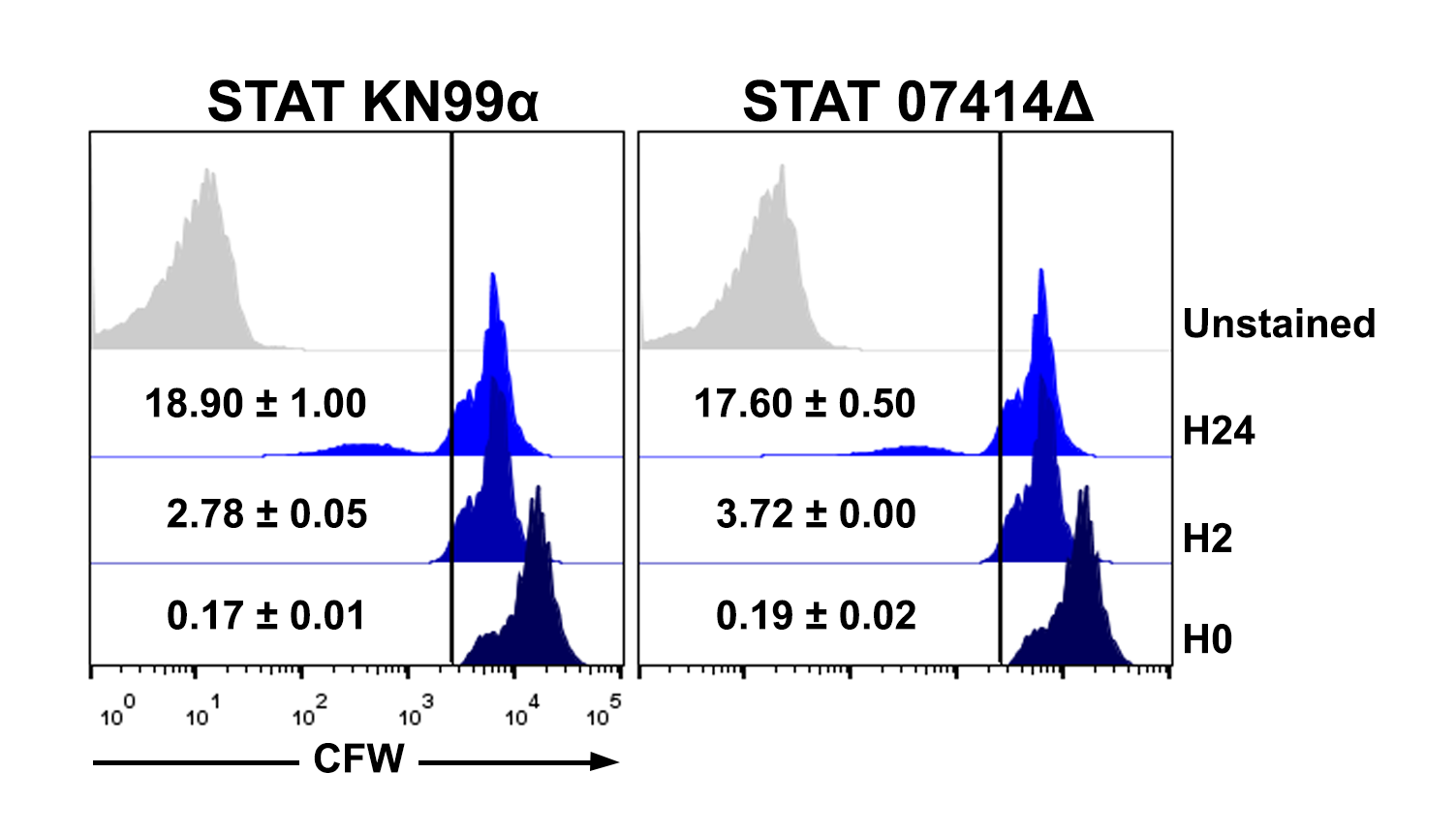

Supplement: S5 Fig — C. neoformans wild-type strain KN99α and 07414Δ mutant cells were labeled with calcofluor white (CFW), opsonized, and incubated with LPS-primed BMDM cells (MOI 2.5) in RPMI + 10% FBS. Yeast cells retrieved from the macrophage upon cell lysis at 2 and 24 h of interaction were analyzed by flow cytometry. The percentage of KN99α and 07414Δ cells exhibiting a medium to low CFW signal (indicative of cells that have undergone proliferation) increased in a comparable manner over time, indicating the presence of PA/PA derivatives in the C. neoformans-containing phagolysosome. Data are means ± SD from 1 representative experiment out of 2. (TIF) [file ppat.1011841.s005.tif]

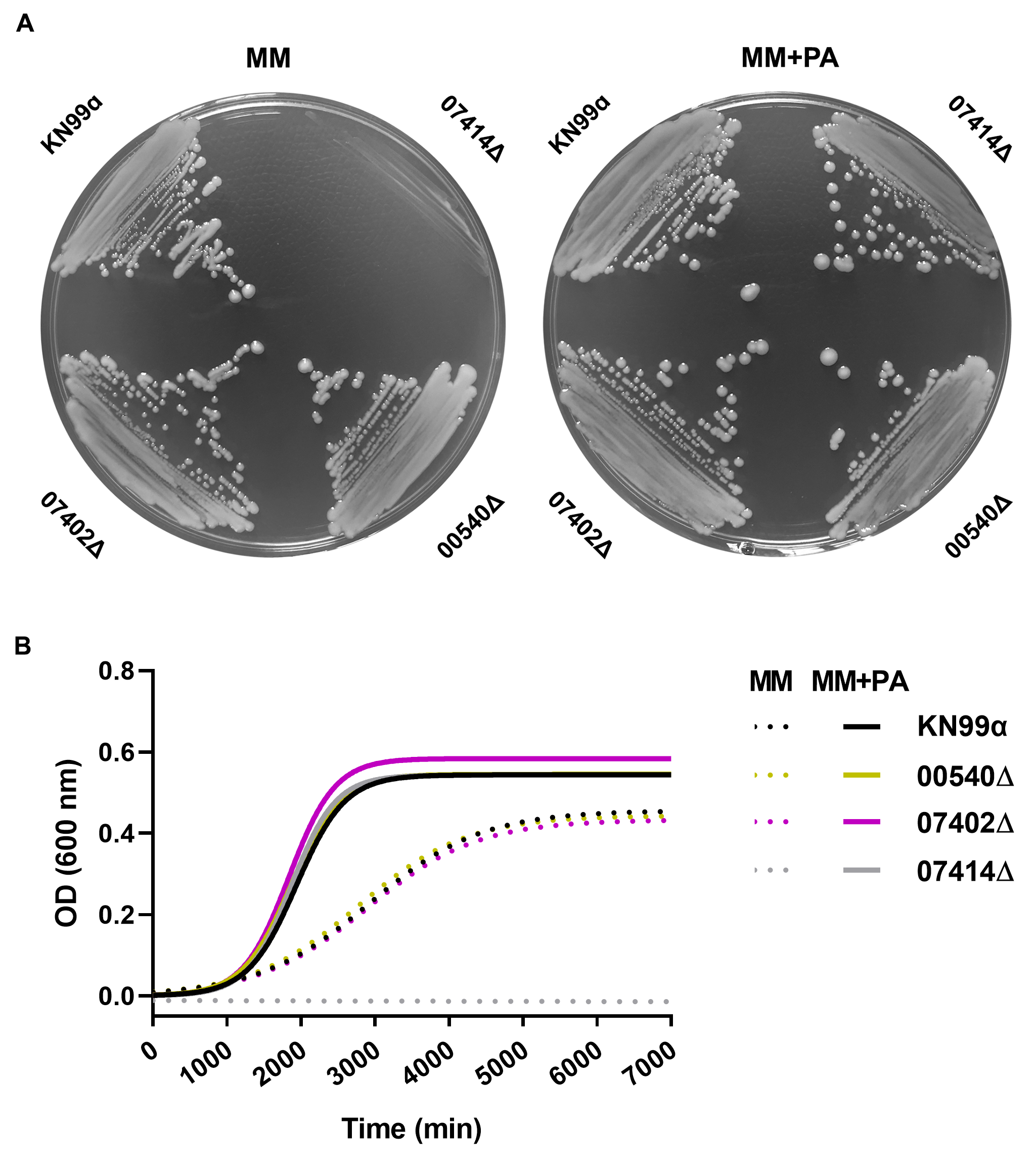

Supplement: S6 Fig — (A, B) The C. neoformans 07414Δ mutant strain, lacking PA synthase, and the 00540Δ and 07402Δ mutant strains (all obtained from Madhani collection), which lack PA transporter proteins, were incubated in minimal medium (MM) in the presence or absence of PA (125 μM). Addition of PA enabled the growth of the 07414Δ mutant, whereas it increased the growth of 00540 Δ and CNAG_07402 Δ mutants similarly to the wild-type strain (KN99α). Data are means from 1 representative experiment out of 2, conducted with 3 technical replicates each. (TIF) [file ppat.1011841.s006.tif]

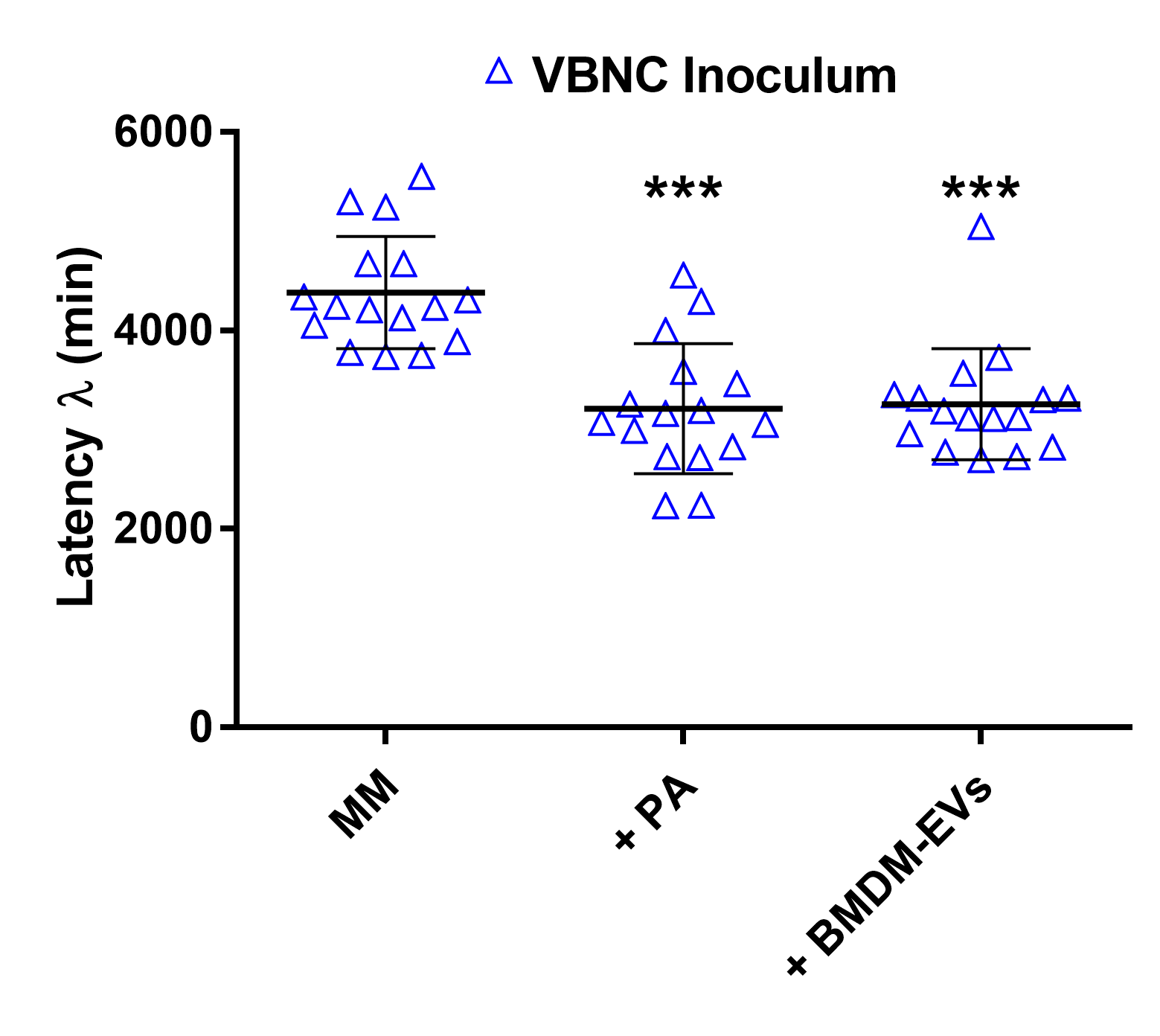

Supplement: S7 Fig — Latency of growth of VBNC cells incubated in minimal medium (MM) is decreased in the presence of pantothenic acid (PA; 125 μM) or EVs (10 μg/mL) isolated from cultures of resting BMDMs. Data are means ± SD from biological duplicates where each dot depicts 1 technical replicate. ***p<0.001, compared to control group (MM). (TIF) [file ppat.1011841.s007.tif]
