# Supplementary material for: Kicking sleepers out of bed: Macrophages promote reactivation of dormant Cryptococcus neoformans by extracellular vesicle release and non-lytic exocytosis
Source: PLoS Pathog. 2023 Nov 30;19(11):e1011841. doi: 10.1371/journal.ppat.1011841 (PMC10715671; doi:10.1371/journal.ppat.1011841)
Supplement: Attachment — Submitted filename: Plos_Pathogens_Reply_20231018.docx [file ppat.1011841.s011.docx]

Translational Mycology group

Department of Mycology

Bat. Duclaux, Aile Fourneau

25, rue du Dr Roux

75015 Paris, France

Tel. +33140613255

Email. dartis@mail.med.upenn.edu

Paris, June 16^th^, 2023

PLOS Pathogens Journal,

Editorial Office

Dear Dr. Kasturi Haldar, Dear Michal Olszewski,

We would like to submit the revised version of our manuscript entitled “Kicking Sleepers Out of Bed: Macrophages Promote Reactivation of Dormant *Cryptococcus neoformans* by Extracellular Vesicle Release and Non-lytic Exocytosis”, by Raffael Júnio Araújo de Castro, Clara Luna Marina, Aude Sturny-Leclère, Christian Hoffmann, Pedro Henrique Bürgel, Sarah Sze Wah Wong, Vishukumar Aimanianda, Hugo Varet, Ruchi Agrawal, Anamélia Lorenzetti Bocca and Alexandre Alanio for evaluation in PLoS Pathogens Journal.

We added a co-author, who was in charge of the sequencing and first analysis of the RNASeq data. He also was in charge of the submissionof the data to the GEO portal.

We really wanted to thank the reviewers for their comments and we clearly have currently a much-improved version of our manuscript expanding the implication of EVs on Cn reactivation to human EVs in addition to mouse EVs.

Regarding the precise role of PA, we softened our conclusions and acknowledge that to reply precisely to the orle of PA in the reactivation in macrophage we would need to engage several months even years of research. Indesed PA is present everywhere in mammalian cells as an essential cofactor making very difficult to study its role in details.

Thank you for considering our rrevised manuscript for publication in PLOS Pathogens Journal,

Yours sincerely,

Raffael Júnio Araújo de Castro

and

Prof. Alexandre Alanio, MD, PhD

Institut Pasteur ; Head of the translational mycology group, Université Paris Cité,

Deputy Director of the National reference center for mycoses and Antifungals

Head of the mycology parasitology laboratory, Saint-Louis Hospital, Assistance Publique Hôpitaux de Paris (APHP), Paris, France.

alexandre.alanio@pasteur.fr

**REPLIES**

Both reviewers appreciated the importance of this work and the quality of the data in most parts. However, some areas of these studies could be strengthened to solidify some of the conclusions.
Whenever conclusion cannot be fully justified with additional data, please soften your conclusions and address the limitations of the current approach.
We are looking forward to seeing the revised manuscript.


Reviewer's Responses to Questions

**Part I - Summary**
Please use this section to discuss strengths/weaknesses of study, novelty/significance, general execution and scholarship.

Reviewer #1: The manuscript takes a very interesting and important look at how macrophages may promote reactivation of dormant Cryptococcus neoformans. Reactivation of dormant C. neoformans infections is certainly a very important topic of clinical relevance. Interestingly, the manuscript also provides some interesting information regarding the impact of introducing yeast at the logarithmic or stationary phases along the growth curve on the macrophage response (not the point of the study but interesting, nonetheless). The studies are, overall, well-conceived and have good controls. The VBNC cell model system for investigating dormancy in C. neoformans is good. The studies show that VBNC cells (and yeast grown to the stationary or logarithmic stage) do not induce much, if any, immunostimulatory responses from macrophages. Also, the data suggests that EVs from resting BMDMs may induce reactivation of VBNC cells. A potential weakness of the study is that the observations were limited to BMDMs. Some data showing similar observations using primary murine monocytes/macrophages and human monocytes/macrophages would have increased the relevancy of the study (although one would not expect for all of the studies to be replicated in another cell type). Also, there is some question as to if the model provided in Figure 9D is fully supported by the results provided in the manuscript (perhaps the model can be revised, more results provided, or this reviewer is mistaken). Overall, this is a nice and thought-provoking study.

Reviewer #2: This manuscript is focused on elucidating the mechanisms that allow for reactivation of dormant or viable, but not culturable (VBNC) C. neoformans cells in the context of macrophage phagocytosis. Using the C. neoformans strain H99O, the authors employ a series of in vitro studies to explore the interplay between VBNC cells and macrophages. Their major findings are as follows: 1) VBNC cells are minimally immunostimulatory in bone marrow derived macrophages (BMDMs) both transcriptionally and via cytokine profiling, 2) VBNC cells are not able to activate the NLRP2 inflammasome, and 3) resting and M2-polarized macrophages can induce reactivation of VBNC cells either through extracellular vesicles of non-lytic exocytosis. The experiments focused on understanding how the macrophage, or host, might benefit from VBNC cell expulsion and how it "reactivates" VBNCs are well conducted with appropriate controls.

**Part II – Major Issues: Key Experiments Required for Acceptance**
Please use this section to detail the key new experiments or modifications of existing experiments that should be absolutely required to validate study conclusions.

Generally, there should be no more than 3 such required experiments or major modifications for a "Major Revision" recommendation. If more than 3 experiments are necessary to validate the study conclusions, then you are encouraged to recommend "Reject".

Reviewer #1: 1. Confirm robustness and human relevancy of observations using primary murine monocytes/macrophages, and human macrophage-like cell lines or PBMCs; respectively. There is no data regarding the promotion of reactivation primary murine cells or human macrophages nor the impact of EVs derived from these cell sources on reactivation.

**REPLY:** In most of the experiments, we used murine bone marrow-derived macrophages (BMDMs), which are primary cells *ex vivo* differentiated from stem cells (Marim *et al.,* 2010). BMDMs are widely recognized and accepted as a suitable model for studying *C. neoformans*-host interactions, as bone marrow-derived infiltrating monocytes account for most of the immune cells engaged in pulmonary immune response against *C. neoformans* infection (Osterholzer *et al.*, 2011). As an exception, we employed J774 murine macrophage-like cell line in **Fig 7F-G** due to its higher permissiveness for *C. neoformans* proliferation, which would facilitate our analysis.

**ACTION 1:** To address the reviewer's requirement regarding the use of primary cells, we now included supplementary data (**S5 Fig**) which reproduces the experiment and the results from **Fig 7F-G** but based on the use of murine BMDMs. This supplementary data demonstrates that the pantothenate synthase mutant proliferates within the BMDM phagolysosome in a comparable manner to the wild-type strain (described in lines 362-364).

**ACTION 2:** To validate the robustness and human relevancy of our experiments demonstrating reactivation promoted by BMDM-EVs, we isolated EVs from murine peritoneal macrophages, which are terminally differentiated macrophages (i.e., matured *in vivo*) obtained from mice peritoneum, as well as from the human monocytic cell line THP-1. We then assessed the impact of these EVs from different cell types on the reactivation of VBNC cells (**Fig 9C**). While we observed a lower capacity in reactivating VBNC cells compared to BMDM-EVs, both cell types produced EVs leading to a decrease in latency which is our readout for reactivation, and in a similar manner. These results reinforce the robustness and human relevancy of our findings obtained with BMDM-EVs.

**ACTION 3:** These new findings are elaborated upon and discussed in lines 437-443 and 611-614, respectively. Of note, since these new results incorporate the conclusions drawn from the initially submitted **Fig 9C**, the latter has been replaced and is now included as a supplementary figure (**S7 Fig**).

2. Question remains if exposure to EVs of M1 macrophages allow for reactivation of C. neoformans or is comparable to exposure to EVs from M0 or M2 polarized BMDMs. The studies in figure 9c were done with EVs from non-polarized BMDMs.

**ACTION 1:** We thank the reviewer for this suggestion. To address this question, we have isolated EVs from M0, M1 and M2 BMDMs to test the influence of macrophage polarization on the reactivation capacity of EVs over VBNCs. Notably, we observed no significant differences among EVs derived from M0, M1, and M2 cells, indicating their similar activities (**Fig 9C**).

**ACTION 2:** These results showed in new **Fig 9C** are described and discussed in lines 434-437 and 649-652, respectively.

3. There were a couple of conclusions described within the proposed model (Figure 9D) that were perhaps not supported by the data shown (or I could have missed it). One is that macrophages can promote induction and maintenance of the dormant state in Cn within the phagolysosome irrespective of the macrophage polarization phenotype (Do the results provided in Figure 6A show that this is not the case with M1 polarized macrophages?). Secondly, the model states that VBNC cells non-lytic exocytosed from MO and M2 cells, but not M1 cells, are prone to reactivation upon exposure to EVs derived from macrophages (I don't think that this was tested using cells exocytosed from M0, M1, or M2 polarized macrophages - perhaps just inferred).

**REPLY:** As demonstrated previously in Alanio *et al*., mBio 2015, macrophages, specifically J774 cells, can promote induction of the dormant state in *C. neoformans* upon phagocytosis of stationary-phase cells. Our present results obtained using an inoculum already induced as dormant/VBNC cells, indicates that dormancy is maintained during internalization by M0, M2 and also M1 BMDMs (**Fig 6C**), as evidenced by the absence of an increase in the CFU of cells retrieved from inside these phagocytes, associated with a limited decrease in yeast viability (**Fig 6D**). **Fig 9D** represents the conclusions of our study in the context of previous research on the induction of dormancy in *C. neoformans* by macrophages (including Alanio *et al*., 2015, mBio and the current work).

In parallel, **Fig 8A** clearly shows that classic M1 macrophages prevent extracellular reactivation of VBNC *C. neoformans*. This was specifically depicted in **Fig 9D** with M1 macrophage inhibiting the reactivation. Our revised model in **Fig 9D** is in line with our revised results.

**ACTION 1:** In addressing the second point, considering also reviewer #2 request, we have revised the model presented in **Fig 9D**, as well as its corresponding legend and throughout the manuscript. The updated model illustrates that both factors, namely extracellular vesicles and non-lytic exocytosis, play a role in promoting reactivation, as demonstrated by the results from **Fig 8** and **Fig 9**. We have revised throughout the manuscript (lines 58-61, 145-147, 409-410, 443-444 and 597-599) to rewrite sentences that were indicating that both factors act together or depend on each other.

Reviewer #2: The authors also attempt to elucidate how VNBCs are able to maintain such a low profile in macrophages using cell wall analysis, as well as pantothenic acid limitation studies. While interesting, I would argue that these experiments on the pathogen-side are superficial and require additional work to fully develop this aspect of the manuscript.

The use of the 0747delta mutant strain to detect the presence of pantothenic acid (PA) is questionable. The authors do not provide data or evidence that this mutant strain is NOT utilizing an alternative biosynthetic pathway or nutrient to maintain viability within the phagolysosome. In other words, given that C. neoformans is known to utilize alternative nutrients to maintain viability, it's ability to grow in the phagolysosome (a historically PA-poor environment) is not surprising as the phagolysosome may contain an alternative nutrient source that C. neoformans may be utilizing instead.

**REPLY 1:** It is a well-founded consensus that *C. neoformans*’ polysaccharide capsule is its main virulence determinant, and also main factor enabling the yeast to maintain a low profile during interactions with phagocytes (Casadevall et al, 2019). Moreover, we had previously characterized the capsule of VBNCs (Hommel *et al*., 2019) (commented on line 497). A significant reduction in median capsule thickness (20%) was observed in VBNCs compared to stationary-phase cells, as measured using transmission electron microscopy. However, VBNC’s capsule displayed consistent staining and distribution/shape, comparable to stationary-phase cells, using the opsonizing antibody anti-glucuronoxylomannan, E1 (Dromer *et al*. 1987), coupled to flow cytometry and fluorescence microscopy. This indicates a robust structural preservation, despite a modest reduction in thickness, of the capsule in the VBNC state.

In the current study, as indirect evidence of the functional preservation of this structure, we found that opsonization of VBNCs with 18B7 (an analogue of E1) resulted in phagocytosis rates as high as those observed in stationary-phase cells (**Fig 8C**). Furthermore, we demonstrated that, apart from the structural aspects, the low metabolism of VBNC also plays a role in maintaining their discrete profile. This was indicated by the inability of VBNC cells, similar to heat-killed stationary-phase cells, to trigger NLRP3-dependent IL-1beta secretion, a response observed in metabolically active (live) stationary and logarithm phase cells (**Fig 3E**). In addition, both the RNAseq and cytokine profiling data emphasize the importance of the yeast metabolism: the lower the yeast cell metabolism, the lower its immunostimulatory capacity. Therefore, the cell wall analysis provided important, albeit complementary, data. Taken together, we have characterized *C. neoformans*’ ability to maintain a low profile using diverse approaches, including biochemical, structural, immunolabeling and metabolic perspectives.

**REPLY 2:** In regards to the studies on the pantothenic acid (PA), we agree with the reviewer’s comment and acknowledge the possibility that *C. neoformans* could be using other sources than PA. It is important to emphasize that PA (or its derivatives) have long been established as vital components in yeasts (Williams *et al.*, 1943; Olzhausen *et al.*, 2009; De Villiers *et al*., 2015) and in mammalian cells. It is present in virtually all the compartments of cytoplasm, including lysosomes (Abu-Remaileh *et al*., 2017), and is used as an essential component in all cell culture media and serum. Mammalian cells rely on the import of PA through many transporters that are essential for cell survival. PA is commonly obtained from the food and microbiota of the gut (Yu *et al.*, 2022). Indeed, fungi including *Cryptococcus* and bacteria are known to be able to produce it by themselves relying on precursor amino acids and not only on the import of PA (Olzhausen *et al.*, 2009; Albuquerque *et al.*, 2014).

We observed that the mutant CNAG_07474, deficient in pantothenate synthase gene, is unable to grow in the absence of PA (**S4 Fig** and **S6 Fig**). Growth in minimal media is abolished in the absence of PA, meaning that this mutant is auxotrophic for PA. The use of auxotrophic strains can be considered the perfect biosensor to detect nutrient availability in complex biological systems (Berney & Berney-Meyer, 2017). So, we used this mutant as a probe for PA bioavailability.

**ACTION 1:** We have corroborated this by incubating 07414∆ mutant strain with a set of nutrients and several key intermediates in metabolic pathways employed by *C. neoformans* during macrophage infection (Alden *et al*., 2022). As shown in the new **S4 Fig** and described in lines 345-348, no growth was observed in the absence of PA.

**ACTION 2:** We used other available mutants, specifically single-gene deletion mutants of PA transporters (00540delta and 07402delta lacking respective PA transporters; from the Madhani collection) to detect the presence or absence of PA in *C. neoformans*-containing phagolysosome. However, these genes, encoding PA transporter proteins, seem to functionally overlap, as evidenced by the enhanced growth obtained in these mutants upon PA supplementation (**S6 Fig**; described in lines 367-369). A co-culture assay conducted with J774 cells and the aforementioned mutants reinforced this observation, revealing that the PA transporter mutants displayed intracellular growth patterns similar (00540delta) or even significantly higher (07402delta) to those of the WT strain (figure provided below). Therefore, a double mutant (00540delta07402delta) would be necessary. While interesting, this inquiry falls beyond the scope of the present study. Moreover, it would offer limited additional insights, considering it constitutes another indirect approach.

Proving that other factors are not involved can be considered as a full research proposal and unfortunately, we are not organized to get such project started right now. In addition, we would need to use serum and medium that are PA free, which costs 2000€/liter each, making it totally unaffordable for us right now.

Furthermore, it is crucial to note that pathogens well adapted to the intracellular lifestyle, including *C. neoformans*, can manipulate the micronutrient composition of the phagolysosome to facilitate survival and proliferation (discussed in lines 533-536). For instance, while PA auxotrophic strains of *M. tuberculosis* exhibit virulence attenuation in mouse infection, *Salmonella* PA auxotrophic strains do not (Bacon *et al.*, 1950; Sambandamurthy *et al.*, 2002). Moreover, *C. neoformans* is known to cause phagolysosome-membrane permeabilization, which potentially enables access to PA from the host cell cytosol.

**REPLY 3:** We are confident that the main point of this aspect of the manuscript, whether the phagolysosome inhibits reactivation due to a lack of reactivation stimuli (regardless of whether it involves PA or any PA replacement), has been properly addressed.

**ACTION 2:** As such, we appreciate the reviewer’s feedback, and we have now duly clarified that our conclusion may be limited to the context of the *C. neoformans*-containing phagolysosome (lines 37-38, 370-373, and 531-533). Furthermore, while our conclusions are grounded in proper and established methodology, in light of the growing evidence regarding CoA biosynthesis involving PA derivatives rather than PA itself (De Villiers *et al.*, 2015), we acknowledge the conceivable role of PA derivatives as alternative sources of PA within the phagolysosome (lines 548-556). This revision has been incorporated in lines 37-38, 364-365, and 548-556.

**Part III – Minor Issues: Editorial and Data Presentation Modifications**
Please use this section for editorial suggestions as well as relatively minor modifications of existing data that would enhance clarity.

Reviewer #1: (No Response)

Reviewer #2: 1. Figure 7B, and 7D: please revise the coloring of the PA concentration lines. The different shades of blue are not evident upon reviewing a physical copy of the manuscript.

**ACTION:** **Fig 7B** and **7D** have been duly revised for enhanced clarity. Additionally, heat maps and other figures in the manuscript also have been updated for better visuality, considering a color-blindness-friendly palette.

2. Figure 9D: While useful, the proposed model for VBNC cell interaction with macrophages is not easy to interpret. For instance, it is not clear to me whether the PA in the phagolysosome is intended to inhibit or promote reactivation. In addition, the discussion section suggests that M1 macrophages might be purposefully promoting expulsion of VBNCs to remove dormant cells without triggering reactivation. This M1 vs. M2/M0 nuance is missing in the proposed model. More detail is needed.

**ACTION:** **Fig 9D** and its corresponding legend has been revised accordingly.

3. Line 139-140: I'm not sure what the authors are trying to state in this sentence.

**ACTION:** The sentence has been revised for better understanding (lines 142-145).

4. Line 204-205: The authors do not specify that the transcriptional data of TNFalpha is not shown in any figures. Please either include this data into a pre-existing figure for reference, or provide language that makes this more clear.

**ACTION:** We have included this data into **S1 Fig** and clarified its corresponding description (lines 209-211).

5. Line 236-239: The VBNC cells cannot induce IL-1b in any mouse lineage, but unclear if VBNCs also do not induce IL-1b in LPS-primed BMDMs.

**ACTION:** We have rephrased this sentence for better clarity (lines 238-243).

6. Line 275: While previous studies show NO production is effective for killing of Cryptococcus, it is unclear to this reviewer that there is a role for NO production in Cryptococcus containment?

**REPLY:** The nitrosative stress elicited by NO has a significant impact on *C. neoformans* cells, including in terms of cell metabolism (Missal *et al.*, 2006). As a consequence, the NO produced by macrophages can exert fungistatic and/or fungicidal activity against *C. neoformans* (Missal *et al.*, 2006, Davis et al, 2013, Leopold-Wagner *et al.*, 2015). We employed the term “contain/containment” to describe the fungistatic capacity of NO on *C. neoformans*.

**ACTION:** We have replaced “contain” with “control the growth” (line 279) to better convey this concept.

7. Line 400-401: Are the authors suggesting EVs from M1 macrophages enhance reactivation at a similar rate to M2/M0 macrophages?

**REPLY:** As indicated, we are specifically referring to the results from **Fig 9B**, which were obtained using M0 macrophages only. Furthermore, the results depicted in the new **Fig 9C** demonstrate that EVs isolated from M1 cells are indeed capable of enhancing the reactivation of VBNC cells in a similar manner to M2/M0 cells. However, as demonstrated in **Fig 8A**, M1 cells are less capable of reactivating VBNC cells compared to M2/M0 in macrophage-VBNC co-cultures. This suggests that the direct interaction with M1 cells may inhibit the ability of M1-EVs to facilitate VBNC cell reactivation.

**ACTION 1:** We have clarified this sentence in lines 420-422.

**ACTION 2:** We have described and discussed the results addressing the question of whether EVs from M1 macrophages enhance reactivation at a similar rate to M2/M0 macrophages in lines 436-437 and 649-652.

**REFERENCES**

Abu-Remaileh, M., Wyant, G. A., Kim, C., Laqtom, N. N., Abbasi, M., Chan, S. H., ... & Sabatini, D. M. (2017). Lysosomal metabolomics reveals V-ATPase-and mTOR-dependent regulation of amino acid efflux from lysosomes. Science, 358(6364), 807-813.

Alanio, A., Vernel-Pauillac, F., Sturny-Leclère, A., & Dromer, F. (2015). *Cryptococcus neoformans* host adaptation: toward biological evidence of dormancy. *MBio*, *6*(2), 10-1128.

Albuquerque, P., Nicola, A. M., Nieves, E., Paes, H. C., Williamson, P. R., Silva-Pereira, I., & Casadevall, A. (2014). Quorum sensing-mediated, cell density-dependent regulation of growth and virulence in *Cryptococcus neoformans*. MBio, 5(1), 10-1128.

Alden, K. M., Jezewski, A. J., Beattie, S. R., Fox III, D., & Krysan, D. J. (2022). Genetic interaction analysis reveals that Cryptococcus neoformans utilizes multiple acetyl-CoA-generating pathways during infection. *Mbio*, *13*(4), e01279-22.

Bacon, G. A., Burrows, T. W., & Yates, M. (1950). The effects of biochemical mutation on the virulence of Bacterium typhosum: the virulence of mutants. *British journal of experimental pathology*, *31*(6), 714.

Berney, M., & Berney-Meyer, L. (2017). *Mycobacterium tuberculosis* in the face of host-imposed nutrient limitation. Microbiology spectrum, 5(3), 10-1128.

Casadevall, A., Coelho, C., Cordero, R. J., Dragotakes, Q., Jung, E., Vij, R., & Wear, M. P. (2019). The capsule of *Cryptococcus neoformans*. Virulence, 10(1), 822-831.

Davis, M. J., Tsang, T. M., Qiu, Y., Dayrit, J. K., Freij, J. B., Huffnagle, G. B., & Olszewski, M. A. (2013). Macrophage M1/M2 polarization dynamically adapts to changes in cytokine microenvironments in *Cryptococcus neoformans* infection. *MBio*, *4*(3), e00264-13.

De Villiers, M., & Strauss, E. (2015). Jump-starting CoA biosynthesis. *Nature chemical biology*, *11*(10), 757-758.

Dromer, F., Charreire, J., Contrepois, A., Carbon, C., & Yeni, P. (1987). Protection of mice against experimental cryptococcosis by anti-*Cryptococcus neoformans* monoclonal antibody. Infection and immunity, 55(3), 749-752.

Hommel, B., Sturny-Leclère, A., Volant, S., Veluppillai, N., Duchateau, M., Yu, C. H., ... & Alanio, A. (2019). *Cryptococcus neoformans* resists to drastic conditions by switching to viable but non-culturable cell phenotype. *PLoS pathogens*, *15*(7), e1007945.

Leopold Wager, C. M., Hole, C. R., Wozniak, K. L., Olszewski, M. A., Mueller, M., & Wormley Jr, F. L. (2015). STAT1 signaling within macrophages is required for antifungal activity against *Cryptococcus neoformans*. *Infection and immunity*, *83*(12), 4513-4527.

Marim, F. M., Silveira, T. N., Lima Jr, D. S., & Zamboni, D. S. (2010). A method for generation of bone marrow-derived macrophages from cryopreserved mouse bone marrow cells. *PloS one*, *5*(12), e15263.

Missall, T. A., Pusateri, M. E., Donlin, M. J., Chambers, K. T., Corbett, J. A., & Lodge, J. K. (2006). Posttranslational, translational, and transcriptional responses to nitric oxide stress in *Cryptococcus neoformans*: implications for virulence. *Eukaryotic cell*, *5*(3), 518-529.

Olzhausen, J., Schübbe, S., & Schüller, H. J. (2009). Genetic analysis of coenzyme A biosynthesis in the yeast *Saccharomyces cerevisiae*: identification of a conditional mutation in the pantothenate kinase gene CAB1. Current genetics, 55, 163-173.

Osterholzer, J. J., Chen, G. H., Olszewski, M. A., Zhang, Y. M., Curtis, J. L., Huffnagle, G. B., & Toews, G. B. (2011). Chemokine receptor 2-mediated accumulation of fungicidal exudate macrophages in mice that clear cryptococcal lung infection. *The American journal of pathology*, *178*(1), 198-211.

Sambandamurthy, V. K., Wang, X., Chen, B., Russell, R. G., Derrick, S., Collins, F. M., ... & Jacobs Jr, W. R. (2002). A pantothenate auxotroph of Mycobacterium tuberculosis is highly attenuated and protects mice against tuberculosis. *Nature medicine*, *8*(10), 1171-1174.

Williams, R. J. (1943). The chemistry and biochemistry of pantothenic acid. *Advances in Enzymology and Related Areas of Molecular Biology*, *3*, 253-287.

Yu, Y., van der Zwaag, M., Wedman, J. J., Permentier, H., Plomp, N., Jia, X., ... & Sibon, O. C. (2022). Coenzyme A precursors flow from mother to zygote and from microbiome to host. Molecular Cell, 82(14), 2650-2665.
